# Application of Cardio-Forecasting for Evaluation of Human—Operator Performance

**DOI:** 10.3390/ijerph17010326

**Published:** 2020-01-02

**Authors:** Anton Panda, Volodymyr Nahornyi, Jan Valíček, Marta Harničárová, Iveta Pandová, Cristina Borzan, Samuel Cehelský, Lukáš Androvič, Hakan Tozan, Milena Kušnerová

**Affiliations:** 1 Department of Automobile and Manufacturing Technologies, Faculty of Manufacturing Technologies with a Seat in Prešov, Technical University of Kosice, Bayerova 1, 080 01 Prešov, Slovakia; anton.panda@tuke.sk (A.P.); iveta.pandova@tuke.sk (I.P.); scehel@gmail.com (S.C.); agent.anim@gmail.com (L.A.); 2 Department of Computer Science, Faculty of Electronics and Information Technologies, Sumy State University, Rimsky-Korsakov, 2, 44007 Sumy, Ukraine; v.nahornyi@cs.sumdu.edu.ua; 3 Department of Electrical Engineering, Faculty of Engineering, Slovak University of Agriculture in Nitra, Automation and Informatics, Trieda Andreja Hlinku 609/2, 949 76 Nitra, Slovakia; jan.valicek@uniag.sk; 4 Department of Mechanical Engineering, Faculty of Technology, Institute of Technology and Business in České Budějovice, Okružní 10, 370 01 České Budějovice, Czech Republic; kusnerova.milena@mail.vstecb.cz; 5 Department of Public Health and Management, Iuliu Hatieganu University of Medicine and Pharmacy, Strada Victor Babeș 8, 400000 Cluj-Napoca, Romania; borzancristina@yahoo.com; 6 Department of Industrial Engineering, School of Engineering and Natural Sciences, Istanbul Medipol University, Kavacik Mh. Ekinciler Cd., 34010 Istanbul, Turkey; htozan@medipol.edu.tr; 7 Health Systems and Policies Research Center, Istanbul Medipol University, Kavacik Mh. Ekinciler Cd., 34010 Istanbul, Turkey

**Keywords:** individual limit of working capacity, trend of heart rate amplitude, trend model, identification of model parameters, cardiogram, model coefficients

## Abstract

The paper presents the results of the development of the cardio-forecasting technology, which introduces a new method to monitor the state of human-operator, which is characteristic for the given production conditions and for individual operators, to predict the moment of exhaustion of his/her working capacity. The work aims to demonstrate the unique, distinctive features of the cardio-forecasting technology for predicting an individual limit of his/her working capacity for each person. A unique methodology for predicting individually for each person the moment when he/she reaches the limit of his/her working capacity is based on a spectral analysis of a human phonocardiogram in order to isolate the frequency component located at the heart contraction frequency. The trend of the amplitude of this component is approximated by its model; consequently, the coefficients of the trend model are determined. They include the operator’s operating time until his/her working capacity is exhausted. A methodology for predicting the moment when he/she reaches the limit of his/her working capacity for each person individually and assessment based on this degree of criticality of their condition will be realized as a software application for smartphones using the Android operating system.

## 1. Introduction

The field of research whose results are presented in the article is limited by the methods of monitoring the health of a human-operator. With the development of technology, physical and, especially, emotional loads acting on a human operator are rapidly increasing [1,2]. As a result, the human operator is increasingly becoming the cause of accidents and adversities with severe consequences, being the weak link of the “man–machine” system [3,4]. Moreover, the moment of exhaustion of the operator’s working capacity must be foreseen in advance, preferably immediately before the start of his/her official duties [5,6]. To date, there are practically no systems for assessing and predicting changes in the functional state of a human operator in the process of professional activity [7,8].

The following methods became frequently used in practice [9,10,11,12,13]:

- questionnaire forms, allowing to obtain a self-assessment of the psychological state of the operator;
- psycho-physiological tests, such as “Determining the critical frequency of flicker fusion”;
- evaluation of electrophysiological indicators, such as heart rate, electroencephalographic indicators, and others;
- study of the biochemical composition of blood and urine.

All the methodologies mentioned above, when used cumulatively, make it possible to obtain an objective assessment of the functional state of a human operator. However, for this assessment, it is necessary to draw the operator away from the work to perform tests and procedures required by the testing methodologies [14]. In order to obtain a picture of the change in the functional state of a human operator during a work shift, the procedure must be repeated at regular intervals. This takes considerable time and, therefore, this approach, although it is the most accurate and reliable, is not applied in practice. Therefore, the efforts of researchers involved in the problem of assessing and predicting changes in the functional state of a human operator in the course of their professional activities are aimed at finding methods that allow obtaining an assessment of the investigated parameters without drawing the operator away from work [15,16].

In this case, the human operator is generally considered to be a system that depletes its resources regardless of its specific type of operation (the type of operator’s work). The aim of the new method of human health monitoring is to predict events and a phenomenon of various origins in connection with Sorett’s forecasting methodology. The essence of Sorett’s methodology is that the required moment when the system reaches the critical state (*t_c_*) is not traditionally determined by the results of the prediction model exceeding the maximum allowable value of the monitored parameter. The *t_c_* parameter is determined here in the process of approximating initial data using their predictive model since it is included as a coefficient directly in its mathematical structure. Consequently, Sorett’s methodology does not require knowledge of traditional statistics on the maximum permissible value of the regulated parameter but relies on an analysis of the nature of the change in the course of the monitored trajectory.

The human body can identify when its limits are exceeded. There are many scientific research papers devoted to human health and monitoring to support human performance. Human factors knowledge is one of the fundamental tools used in the healthcare sector. Human factors are about optimizing the performance of people in the workplace for safety, well-being, and efficiency. They consider the work environment in terms of focusing on people, looking at the whole system and its impact on people’s behavior and common interactions. Human factors play an increasingly important role in modern complex systems where safety is critical because human errors are often the cause of incidents and accidents despite the strict safety culture prevailing in most societies. Fatigue is the key factor of human performance since it allows the human body and cognitive skills to perform at an optimum level [17,18]. Fatigue can be defined as an intense feeling of tiredness or weakness associated with reduced ability to concentrate on performing ordinary tasks. Physically, fatigue and increased fatigability point to a lack of energy, muscle weakness, and deficient functioning of the nervous system [19]. Other words for fatigue can be found in literature as exhaustion, lethargy, languor, or listlessness. Fatigue can be divided as mental or physical. Physical fatigue is defined as overall exhaustion of the whole body (mainly muscle weakness), while mental fatigue describes mainly the effect of mental fatigue on cognitive skills (for example, burn-out syndrome). A general summary of the most common fatigue risk factors is inadequate sleep quality, sleep deprivation, physical and mental exertion, emotional stress, disruption of circadian rhythms, or poor physical condition [19,20]. In any case, monitoring of fatigue is a very complex and important task.

This article presents the main results of the research aiming to develop a method for a quantitative assessment of the individual norm of the functional state of a human operator in the course of professional activity. This assessment relies on the spectral analysis of electrocardiogram records, separation of the spectral component that coincides with the heart rate, a time series of amplitudes of this component, and forecasting based on examination of the nature of changes in this series in the work process of a person till the moment of his/her exhaustion. This fact is the novelty of this research. In order to solve this problem, it is necessary to carry out proactive forecasting of the onset of this event [21,22,23]. This circumstance has served as a serious stimulus for the development of a new forecasting methodology, called “cardio-forecasting”, which is radically different from the existing ones [24,25,26].

## 2. Materials and Methodology

### 2.1. Subject of Research

The subject of research was the recordings of cardiograms of 4 operators, that were taken during the work shift with a duration of 14 h (operators 1 and 2) and within two working weeks with a break for rest (operators 3 and 4).

The task of the authors as specialists in predicting the onset of the critical state of any naturally observed system was to develop a method for predicting the onset of the critical state of the biological system—the human-operator. Customer of the methodology—doctors, provided us with initial data in the form of cardiogram records. The customer divided the data into two groups, the results of long-term and short-term observation. The first was measured in days, the second in hours. The type of employees does not matter. The gradient of intensity (amplitude) of the trajectory of the frequency component of the heart rate spectrum plays a role in the prediction. The originality of the paper provided by the authors:

- acquired heart rate spectrum;
- the spectrum shows the baseline (harmonic) heart rate;
- a plan for the change in time of the amplitude of this harmonic is drawn up;
- determination of the moment of reaching the limit state by the biological system (human-operator).

### 2.2. Research Procedure

The research procedure carried out to confirm the effectiveness of the use of cardio-forecasting for determining the moment of achievement of fatigue limit by each of the individuals in the given conditions of work that were specific only to him/her, assumes monitoring of the performance capability of a human operator in the online mode during working hours. The principle of the new methodology is not based on statistical evaluation of records of form, size, sequence, and combination of the stress of a specific set of surveyed respondents. Statistics of this type would have a specific validity for stressing selected respondents in carrying out the selected specific activity. However, each respondent or patient presents his or her unmistakable specific data, which may considerably differ from the mean and unusable for a specific prediction of his/her health condition under stress. The measurements were performed by the staff of the Faculty of Medicine at the university (at the Medical Faculty of Sumy State University). The total time of the experiments was two months. The database of the measurements was converted into spreadsheets and evaluated using a computer. The amplitude of cardiac muscle oscillation at the baseline frequency of its contractions (~1 Hz) was considered a control parameter. The Xiaomi MI Band 4 Fitness Bracelet was used. The number of participants is fewer than 33, but the results of the presented study are not based on statistical evaluation and generalization. The recording of pulse oscillations was performed using a fitness bracelet, information from which was transmitted via the Bluetooth system to a smartphone using the Android OS. The software installed in the smartphone subjected the recorded signal to spectral analysis. The sampling rate was 1024 Hz; the number of the read points was 8192 pieces, which provided a frequency step of 0.125 Hz. The results of this processing are stored in the Internet cloud under the name of a human operator. Thus, a database was created to be used for forecasting his/her working capacity. The frequency component was singled out from the spectrum that corresponded to the heart rate (HR). A time series was compiled (a trend of the amplitude of HR) based on the results of the regular amplitude measurement.

### 2.3. Definitions and Criteria

The studies investigated two types of forecasting—short-term and long-term. The short-term forecasting provides a forecast for one or two work shifts, during the long-term forecasting for one or two working weeks.

### 2.4. Forecast Duration of Work

Time series was approximated by a trend model in order to determine its coefficients. From the point of view of mathematics, the approximation consists in minimizing the difference between the m pairs of calculated values $A_{HR}^{C}$ and actual values $A_{HR}^{ACT}$ of the HR amplitude. These values characterized the person’s fatigue at each of the m moments of checking his/her state as in Equation (1):

(1) $$U=\sum_{i=1}^{m}{\left(A_{HR}^{C}-A_{HR}^{ACT}\right)}^{2}.$$

The minimization of Equation (1) was performed by one of the methods for finding the minimum of the function [1]. The values $A_{HR}^{C}$ were calculated using Equation (2), which was a model of the trend of the amplitude $A_{HR}^{C}$:

(2) $$A_{HR}^{C}=A\cdot {\left(\frac{T_{CR}-t_{0}}{T_{CR}-t}\right)}^{\alpha },$$

where: *T_CR_*—forecast duration of work of the human operator until fatigue begins; *t*_0_, *t*—the duration of work of a human operator, respectively, at the time of the beginning and the current moment of monitoring his/her state; *A*, *α*—coefficient models.

In Equation (2), there is a fractional rational power function that has no mathematical limit of the course with a warning value signaling the need to end the patient’s exertion. The interruption of strenuous activity in Equation (2) must be determined based on the prediction of the moment of exhaustion of the operator’s work capacity, i.e., that the predicted *T_CR_* time of possible critical exertion and the actual measured *t* time of exertion are equal. The course of the performance degradation process is gradual, with deteriorating conditions. The basic principles of the fuzzy set theory can be used to formalize the description of these states [27]. According to this theory, the degree of criticality of the technical state of the product can be estimated using the *a_COST_* linguistic variable, which is called the state indicator of the monitored operator. In particular, this indicator makes it possible to compensate for the impossibility of statistical prediction, especially on the maximum permissible values of the monitored parameters for relatively small sets. The function used to evaluate this indicator converts the values of the input variables characterizing the operation exertion and the individual properties of the monitored operator to the values of the language variables. These language variables are compared to their standard values or terms, which represent diagnoses of the current state of the monitored operator. The method of dimensional analysis, similarity theory and dimension [28] can be used to obtain an analytical expression for the searched function by which a linguistic variable is calculated as the *a_COST_* status indicator. Based on this procedure, the *a_COST_* status indicator can be evaluated by Equation (3):

(3) $$a_{COST}=\frac{A_{HR}^{C}\cdot t}{A_{HR}^{ACT}\cdot T_{CR}}.$$

During operation, however, the state of the operator changes, so the actual value of the *a_COST_* linguistic variable, calculated from the results of the measurement of the regulated parameter and the prediction of the working capacity of the operator under research, also changes. The numerator in Equation (3) is equal to the product of the current signal level and the current operating time, characterizing the degree of the operator’ exertion under his/her operating conditions. The denominator in Equation (3) is equal to the product of the original signal and source level, reflecting the individual characteristics of the monitored operator, his/her working capacity, potential, and it remains practically unchanged during the operation of the operator under research. In principle, therefore, it quantitatively describes the properties (potential) of the operator under research. Equation (3) can also be seen as the product of two fractions in Equation (4), where the first fraction corresponds to the ratio of the height amplitude intensity level and the initial signal level, the second fraction being the ratio of the current operating time to the estimated working capacity exhaustion:

(4) $$a_{COST}=\frac{A_{HR}^{C}}{A_{HR}^{ACT}}\cdot \frac{t}{T_{CR}}$$

If the status indicator as the *a_COST_* linguistic variable is at most equal to 1, an accident occurs. Under real-life conditions ($A_{HR}^{C}$ > $A_{HR}^{ACT}$, *t* < *T_CR_*) must be stopped at the moment when the threshold of 0.9 is exceeded.

The quality (probability) of the forecast was assessed by the results of its comparison with the working time. The stability of the short-term forecast was also considered. The degree of its variability was estimated by the value of the mean-square deviation of forecasting the working capacity (rms). The paper deals with two types of forecasts: short-term and long-term; the first examines a category of workers working for two shifts in a row (cargo drivers for long distances, supermarket workers, etc.), the second one examines continual monitoring of workers, etc. The source materials for forecasting were presented by the company that controlled the state of a human operator in various areas of his/her activity. This methodology is being experimentally tested at the Medical Institute of the Sumy State University (Sumy, Ukraine).

## 3. Results and Discussion

### 3.1. The Source Data for Forecasting

**Figure 1:**
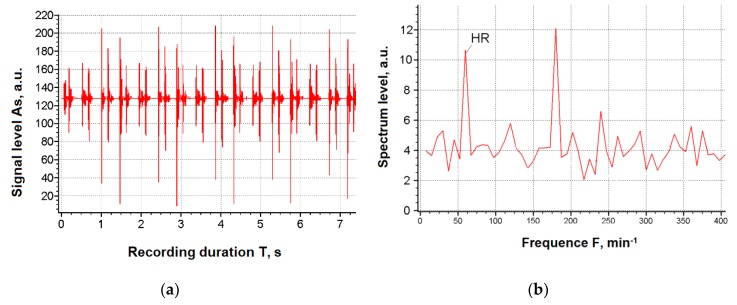
Source data for cardio-forecasting: (**a**) Temporary recording of a signal (cardiogram); (**b**) Spectrum of heartbeats.

Figure 1 shows the typical cardiogram records and their spectra, used as source input data for forecasting the degree of the working capacity of a human operator. The aim of assessing the working capacity of a person is to determine whether physical, psychological, or sensory exertion does not exceed the physiological possibilities of the organism and cannot cause health damage.

In Figure 1b, the HR sign indicates the heart rate, the amplitude of which is a symptom of the degree of criticality of the state of the operator (worker).

### 3.2. Short-Term Forecasting

**Figure 2:**
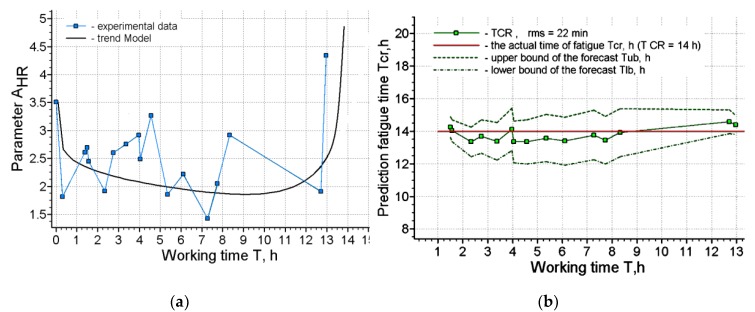
Forecasting results for operator 1: (**a**) Approximation of the source data by the trend model; (**b**) The results of the forecasting.

**Figure 3:**
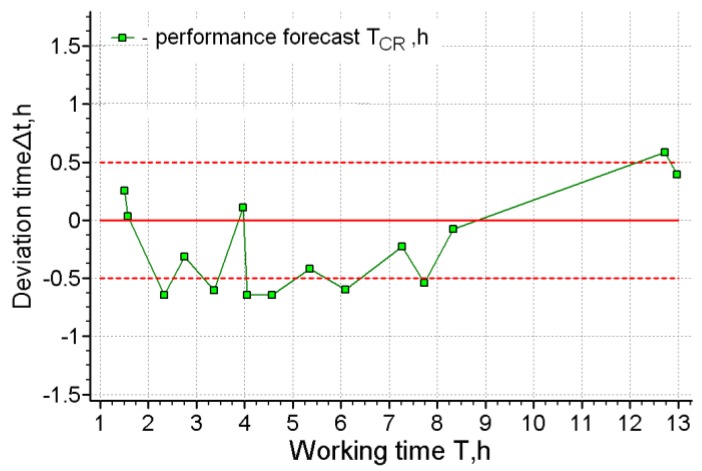
The deviation of the forecasting of working capacity from the actual time of exhaustion for operator 1.

**Figure 4:**
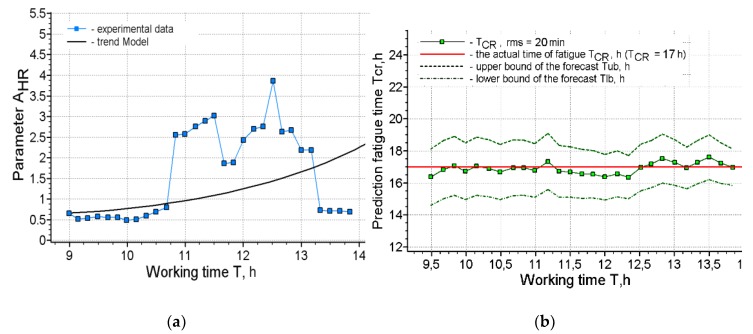
Forecasting results for operator 2: (**a**) Approximation of the source data by the trend model; (**b**) Forecasting results.

**Figure 5:**
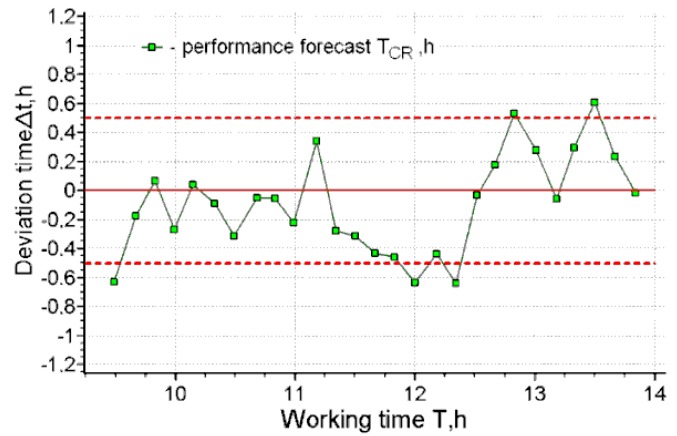
The deviation of the forecasting of the working capacity from the actual time of exhaustion for operator 2.

Figure 2, Figure 3, Figure 4 and Figure 5 present the results of short-term forecasting. The colors in the figures: blue—source data, green—calculation results, black—source data trend. Figure 3; Figure 5 show the deviation of the prediction error. As it follows from Figure 2; Figure 4, the technology of cardio-forecasting makes it possible already at the beginning of the working period (from the 1st to the 2nd hour of work) to consistently predict the time of exhaustion of human working capacity. Moreover, as we can see (Figure 3; Figure 5), the deviation of the forecast from the actual time of exhaustion of working capacity is within 30 min. The variability of the forecast is also insignificant, and it is characterized by the value of rms, varying within 20–22 min (Figure 2b and Figure 4b).

### 3.3. Long-Term Forecasting

**Figure 6:**
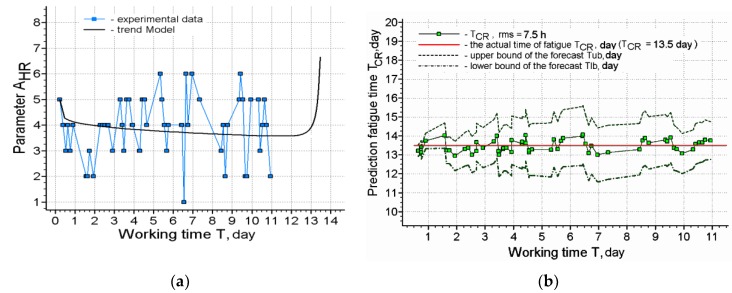
Forecasting results for operator 3: (**a**) Approximation of the source data by the trend model; (**b**) Forecasting results.

**Figure 7:**
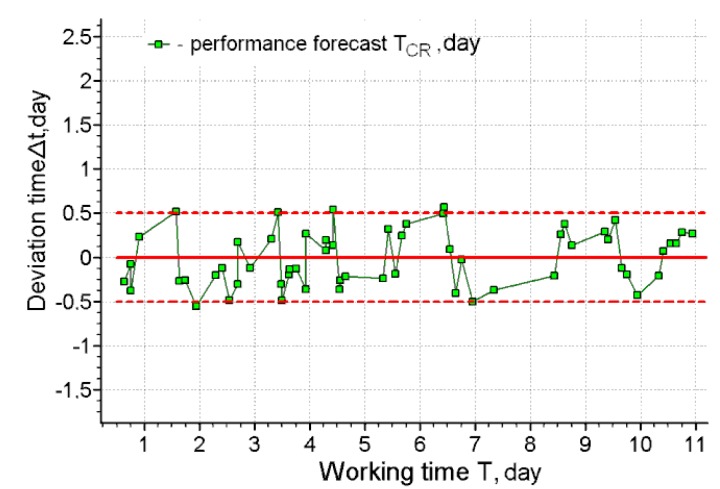
The deviation of the forecasting of the working capacity from the actual time of exhaustion for operator 3.

**Figure 8:**
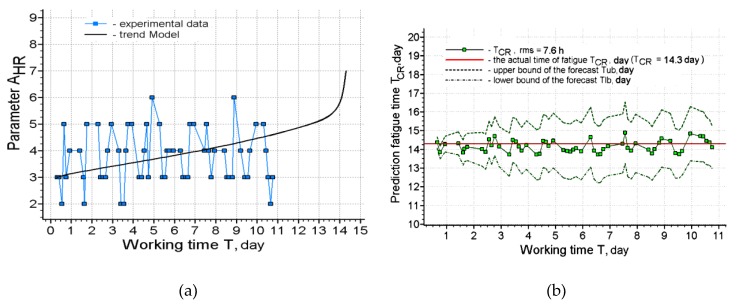
Forecasting results for operator 4: (**a**) Approximation of the source data by the trend model; (**b**) Forecasting results.

**Figure 9:**
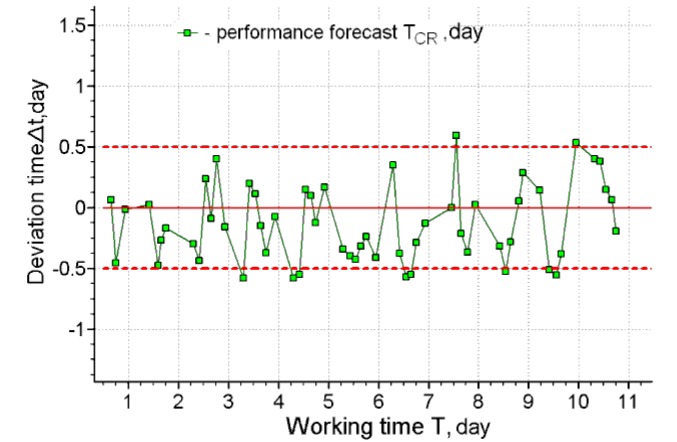
The deviation of the forecasting of the working capacity from the actual time of exhaustion for operator 4.

Figure 6, Figure 7, Figure 8 and Figure 9 present the results of long-term forecasting. As it follows from Figure 6; Figure 8, the use of the cardio-forecasting technology for long-term forecasting makes it possible, already at the beginning of the working period, measured in days, to predict the moment when the working capacity of the human operator is exhausted. At the same time, the deviation of the forecast from the actual time of exhaustion of the working capacity is within half a day (Figure 7; Figure 9). The variability of the forecast is also insignificant, and it is characterized by a rms value not exceeding 7.6 h—( Figure 6b and Figure 8b). Thus, the above results of cardio-forecasting clearly show its advantages compared to the current method of predicting the state of the working capacity of a human operator oriented on the etalon of the working capacity of a human operator.

### 3.4. Analysis and Interpretation of Results

The measurement results in the presented publication are strictly individual, in accordance with the concept of the new methodology applied to the particular monitored patient. The aim of predicting the risk status of this selected patient during stressful activity is to obtain his/her individual data from blood pulse measurement continuously over time. These data characterize the specific performance given by many diverse parameters incomparable to the specific performance of other patients or healthy people. Therefore, this method is not a statistical method but is based on the evaluation of individual trend coefficients of the prediction equation on the basis of fuzzy set theory, dimensional analysis, similarity, and dimension theory. In the context of such predicted values, it is based on the currently measured individual values of the patient’s pulse development time. The particular subject under investigation, i.e., the patient or worker active without rest, may be depleted after 17 h (i.e., up to twice the standard work shift), but with continuous rest, the same worker will be exhausted after 13.5 days at a comparable activity.

In other words: if the rest intervals are longer than that, it will take longer before the worker is exhausted; the length of the intervals in question will be individually different since the fatigue limit is very individual. The fatigue depends mainly on the individual disposition of the monitored operator and the intensity of his/her exertion, in extreme fatigue, it is the case of exhaustion of the organism. Although the character of the fatigue-exhaustion trend is generally valid qualitatively, each worker shows his/her specific characteristics with quantitatively different parameters. The prediction of cardiac activity during fatigue and in a state of extreme exhaustion is a prediction of the moment of exacerbation of the patient’s cardiovascular disease.

The task of the authors as specialists for predicting the onset of the critical state of any naturally observed system (also in engineering or seismology) was to develop a method for predicting the onset of the critical state of the biological system—the human-operator.

The prediction used so far traditionally consists of two phases. The first one is determined on the basis of available data by the parameters of the approximation function. The second function is determined by extrapolating the graph of this function, based on the intersection with the maximum acceptable level of the observed parameter. The intersection coordinate on the x-axis is a desirable argument for a function that determines when the monitored event or phenomenon reaches its maximum allowable (critical) state. Unfortunately, it should be noted that this forecast is not sufficiently reliable. The uncertainty of the traditional method is explained by the fact that the maximum permissible value of the criterion of the monitored parameter is of medium statistical nature. As a result, the average statistical value of the monitored parameter only describes the actual condition of the person with only a certain degree of probability. This circumstance leads to errors in predicting when a particular person reaches a specific (individual) fatigue limit that is only characteristic of the monitored operator and no other subject.

The search and implementation of methods for reliable assessment and prediction of human organic changes, especially in the process of carrying out burdensome professional activities, is a serious scientific and application task in health care. Examples of using this methodology are also given in [25,26].

## 4. Conclusions

The results presented in the publication are entirely original, both the follow-up equations and graphs. The novelty of the cardio-forecasting technology lies in the fact that it does not require knowledge of a statistically unreliable maximum permissible value of the monitored parameter, the achievement of which, according to current methods, indicates the exhaustion of the human working capacity. Therefore, this methodology should be applied to the phenomena for which there are no norms or statistics of their limiting state. At this time in prognostication, a predictive model has been developed; the number of its coefficients is included in a parameter that numerically coincides with the moment when the monitored human health reaches its critical state.

The novelty in experimental techniques allows determining the moment of fatigue reached by people in a wide range of professions—from a surgeon to a train driver or a pilot. Newly, the article showed a change in human heart rate at baseline (~1 Hz) as fatigue develops graphically. The novelty is also that the heart rate is not heard by the doctor (human hear the sound from approximately 20 Hz) and, therefore, indirectly controls the heart’s behavior starting from the 20th harmonic frequency. This methodology was already expected for a long time for the use of fitness bracelets for the rapid assessment of human health. This method should be applied in a stationary, portable and handheld (smartphone) variants in combination with fitness-bracelets.

Potential users can be found in a wide range of human activities and professions from cardiologists to athletes and their coaches, and common people who want to monitor their health. The technology is a distinctly pronounced example of proactive forecasting based on the online tracking of the trend of the monitored parameter (amplitude of the HR frequency spectrum of the electrocardiogram), which makes it possible to anticipate operatively the moment of exhaustion of the working capacity of a human operator long before an onset of a critical state of the given person.

In this regard, the addition of this breakthrough technology to the existing methods of assessing the degree of human working capacity represents a significant advantage over competing methodologies. This is explained by the fact that the existing methods for assessing the state of a person, despite their external diversity, are oriented on comparing the current (static, frozen) physiological portrait of a person with certain etalon of him/her. However, strictly speaking, there is no such reference general etalon for a person as an individual. Figuratively speaking, the etalon is inside the given person; in a certain sense, it is the etalon for him/her.

The technology of cardio-forecasting guided by the requirements adopted in medical history (anamnesis) considers not only the current state of the human body but, at the same time, it takes into account previous events. It uses this internal etalon when forecasting the future condition of a person.

This etalon defines the dynamics of changes in a person’s state that is unique for each person. Externally, this dynamic is manifested as the change in the trend of the amplitude of the frequency component of the HR spectrum of heart fluctuations, which characterizes the change in the human working capacity during the observed period.

## 5. Patents

Patent UA 38438A U Ukraine, Method for determining the machine residual life/Nagornyi V. M. Published on 15 May 2001, Bull. No. 4.

Patent UA 51154 A Ukraine, Vibration diagnostics method of the machine technical condition/Nagornyi V. M. Published on 15 Novober 2002, Bull. No. 11.
